# Towards Molecular Understanding of the Functional Role of UbiJ-UbiK_2_ Complex in Ubiquinone Biosynthesis by Multiscale Molecular Modelling Studies

**DOI:** 10.3390/ijms231810323

**Published:** 2022-09-07

**Authors:** Romain Launay, Elin Teppa, Carla Martins, Sophie S. Abby, Fabien Pierrel, Isabelle André, Jérémy Esque

**Affiliations:** 1 Toulouse Biotechnology Institute, TBI, Université de Toulouse, CNRS, INRAE, INSA, 31077 Toulouse, France; 2 Univ. Grenoble Alpes, CNRS, UMR 5525, VetAgro Sup, Grenoble INP, TIMC, 38000 Grenoble, France

**Keywords:** ubiquinone, MD simulations, Alphafold2, molecular modelling, Martini 3 force field, peripheral membrane protein, co-evolutionary information, protein–protein interaction, umbrella sampling

## Abstract

Ubiquinone (UQ) is a polyisoprenoid lipid found in the membranes of bacteria and eukaryotes. UQ has important roles, notably in respiratory metabolisms which sustain cellular bioenergetics. Most steps of UQ biosynthesis take place in the cytosol of *E. coli* within a multiprotein complex called the Ubi metabolon, that contains five enzymes and two accessory proteins, UbiJ and UbiK. The SCP2 domain of UbiJ was proposed to bind the hydrophobic polyisoprenoid tail of UQ biosynthetic intermediates in the Ubi metabolon. How the newly synthesised UQ might be released in the membrane is currently unknown. In this paper, we focused on better understanding the role of the UbiJ-UbiK_2_ heterotrimer forming part of the metabolon. Given the difficulties to gain functional insights using biophysical techniques, we applied a multiscale molecular modelling approach to study the UbiJ-UbiK_2_ heterotrimer. Our data show that UbiJ-UbiK_2_ interacts closely with the membrane and suggests possible pathways to enable the release of UQ into the membrane. This study highlights the UbiJ-UbiK_2_ complex as the likely interface between the membrane and the enzymes of the Ubi metabolon and supports that the heterotrimer is key to the biosynthesis of UQ_8_ and its release into the membrane of *E. coli*.

## 1. Introduction

Ubiquinone (UQ) is a redox-active prenol localised in cellular membranes of bacteria and eukaryotes [1,2]. By its conservation and its functions, such as an electron shuttle in the respiratory chain or an antioxidant in the reduction of hydroxyl radicals, UQ is a key molecule [3]. UQ is composed of two main parts, a redox-active aromatic group forming the polar head and a hydrophobic polyisoprenoid tail [1]. The redox head can adopt three main redox states: (i) an oxidised form (quinone), (ii) a reduced state (quinol) and (iii) an intermediate form corresponding to a dehydrogenated radical (semi-quinone). The size of the polyisoprenoid tail ranges from 4 to 14 isoprenyl units [4], with this number being species-dependent [5].

**Figure 1:**
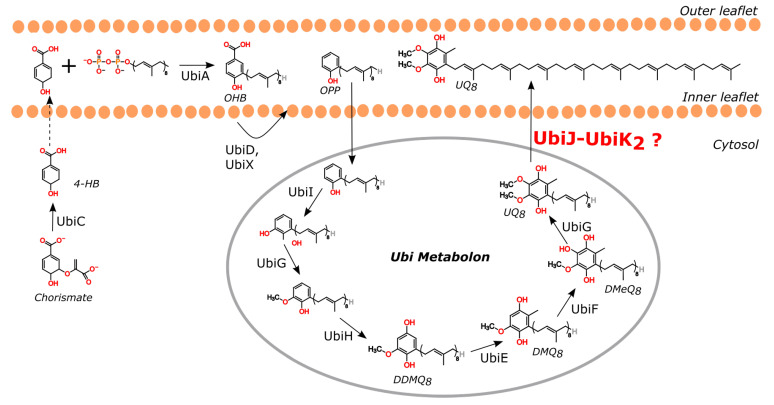
Ubiquinone biosynthesis pathway in *E. coli* supramolecular organisation. Molecules are represented in their reduced forms. Abbreviations: 4-HB: 4-hydroxybenzoate; OHB: octaprenyl-4-hydroxybenzoate; OPP: octaprenyl phenol; DDMQ_8_: C2-demethyl-C6-demethoxy-ubiquinone 8; UQ_8_: Ubiquinone 8.

The quinone head of ubiquinone in bacteria is derived from the shikimate pathway, a seven-step pathway, which converts phosphoenolpyruvate and erythrose-4-phosphate into chorismate [6]. Chorismate is the precursor of aromatic amino acids and many aromatic secondary metabolites, including 4-hydroxybenzoate (4-HB) which is the precursor of the aromatic ring of UQ (Figure 1). Depending on the species, the conversion of chorismate into 4-HB is catalysed either by a chorismate pyruvate-lyase (UbiC) such as in *Escherichia coli* [7], or by a chorismatase called XanB2 [8]. The second component of ubiquinone precursors is the polyisoprenoid tail, which results from the successive action of a farnesyl diphosphate synthase (IspA) and an octaprenyl diphosphate synthase (IspB) that fuse alone with dimethylallyl diphosphate (DMAPP) unit and several isoprenyl-pyrophosphate units. A 4-hydroxybenzoate octaprenyltransferase (UbiA) links the polyisoprenoid tail and 4-HB to produce a ubiquinone precursor, called 3-octaprenyl-4-hydroxybenzoate (OHB) [9]. OHB is decarboxylated by two enzymes UbiD (3-octaprenyl-4-hydroxybenzoate carboxy-lyase) and UbiX (Flavin prenyltransferase). The resulting product is OPP (octaprenyl phenol), which is modified by a series of methylation and hydroxylation to form UQ. These modifications are ensured by a cytosolic complex, called the Ubi metabolon [10]. This multiproteic complex from *E. coli* has been shown to be composed of five enzymes (three hydroxylases and two methyltransferases), but also two structural subunits corresponding to UbiJ and UbiK. Nevertheless, the structural organisation as well as the stoichiometry of the different partners in the metabolon remains to be elucidated. In this biological context, UbiJ and UbiK are thought to play a role in the integrity of the multienzymatic complex, in the binding of UQ biosynthetic intermediates and in the release of the final product UQ, called UQ_8_ in *E. coli*, into the membrane. For these reasons, this work will focus on better understanding at molecular level the role of the two subunits UbiK and UbiJ.

UbiJ is composed of a sterol carrier protein 2 (SCP2) N-ter domain, which is known to bind non-specific lipids and most likely the UQ_8_ molecule. Indeed, the SCP2 domain is a well characterised structural element, known to participate to the lipid storage traffic, cellular signalisation and cellular metabolism [11]. In spite of the diversity of the ligands that have been reported to bind to the proteins of this family, the structure of these proteins is highly conserved, forming a “dome” [11] with a beta-sheet organisation composed of five strands and four alpha-helical elements.

UbiK belongs to the *Brucella* membrane fusogenic protein (BMFP) family, known to interact with the membrane as shown in different studies, but also to be involved in a coiled-coil interaction to form a homotrimer [12,13,14]. Indeed, these previously cited studies have determined the helical propensity in the BMFP family, showing the presence of a coiled-coil region in the C-ter region whereas the folding of the N-ter depends on conditions such as pH, salt concentration or the proximity to the membrane. The N-ter region of the BMFP family could adopt disordered conformations in solution or amphipathic helices when interacting temporarily with the membrane. Interestingly, Carrica et al. have shown that the N-ter region of UbiK, also named YqiC, has a potential role of membrane fusion involved in the pathogen–host interaction in *Salmonella enterica* [14]. Moreover, fractionation studies showed that UbiK in *E. coli* was predominantly associated with the membrane, although a significant fraction of UbiK was detected in the cytosol, such as the other proteins of the Ubi metabolon [10]. In terms of protein interactions, Loiseau et al. have shown that UbiK could form a homotrimer but also an heterotrimer together with UbiJ, referred as UbiJ-UbiK_2_ [12]. This complex was proven by co-expression and co-purification experiments, and its stoichiometry of one UbiJ for two UbiK was determined using a combination of size exclusion chromatography-multi-angle light scattering (SEC-MALS) and analytical ultracentrifugation [12]. Moreover, the authors showed by truncating the N-ter region of UbiJ, that the interaction occurs between the C-ter of UbiJ and UbiK. The molecular association between UbiK and UbiJ is mediated by a coiled-coil interaction in the C-termini, and a preliminary molecular model of UbiJ-UbiK_2_ was proposed [12]. Coiled-coil domains have a high prevalence in biology and are found in a huge diversity of functions [15]. The strength of interaction with coiled-coil domains and their stability are correlated with the size of the interface area [16].

To date, the structural characterisation of UbiJ and UbiK remains very limited. Only the N-terminal region (~120 first residues) of UbiJ, corresponding to the SCP2 domain, has been determined by X-ray crystallography [10] (PDB codes: 6H6O, 6H6N, 6H6P). Moreover, no 3D template is available for a proper modelling of the rest of UbiJ or for the full UbiK. Given these limitations, the modelling of the UbiJ and UbiK molecular complex remains highly challenging. Therefore, the use of adapted methods, such as ab initio methods, to predict the protein structures is required. The development of this type of methods is crucial in the protein modelling field and has been spotlighted by the game changer AlphaFold2 (AF2) [17], outperforming other structure prediction methods in the last CASP challenge [18]. One main reason for the outperformance of ab initio methods is the coupling of (co)-evolutionary information with artificial intelligence algorithms such as machine learning or deep-learning (like in AF2). Indeed, previous work has already demonstrated the advantage of using co-evolutionary information for residue contact prediction [19]. The use of artificial intelligence approaches enables not only to increase performances but also the prediction quality [20]. Interestingly, this development can also be applied for inter-residue contact prediction in the case of protein–protein interactions as shown in the 7th CAPRI challenge [21] and more recently with Alphafold2 multimer [22]. The main limitation of these approaches remains the limited consideration of protein flexibility in the structure prediction. To tackle this issue, molecular dynamics (MD) simulation is a standard and powerful computational method to investigate protein dynamics, to perform conformational sampling and to analyse interactions with other partners (ligand, protein, …) in realistic environments (solvent, membrane, …) [23].

The emergence of AF2 offers the opportunity to revisit the modelling of UbiJ-UbiK_2_ complex previously proposed by Loiseau et al. [12], using methods that have proved to be highly efficient for the modelling of proteins which have limited available structural information. Herein, we used a multiscale modelling approach to investigate first the UbiJ and UbiK in isolated forms, and then the UbiJ-UbiK_2_ heterotrimer. We also studied the interaction with the membrane, the binding of UQ to the UbiJ-UbiK_2_ complex and its release into the membrane using a combination of biased MD simulations and free energy calculations. Together, our results establish UbiJ-UbiK_2_ as the entity that mediates the interaction of the Ubi metabolon with the membrane and strongly suggest that newly synthesised UQ_8_ is released in the membrane directly from the SCP2 domain of UbiJ.

## 2. Results and Discussion

### 2.1. Protein Monomer Modelling

The first step of our study is aimed at modelling UbiJ (Uniprot ID: P0ADP7, 201 amino acids) and UbiK (Uniprot ID: Q46868, 96 amino acids) from *E. coli* in monomeric forms. Given the limited available structural data on these proteins, we opted to use AF2 in order to predict the three-dimensional models of UbiJ and UbiK. By default, five structural models were built for each protein. The quality of 3D models was analysed through the AF2 confidence score, named plDDT (predicted local-distance difference test), adapted from a score for comparison between model and structure of protein, the lDDT score [24], which ranges from 0 to 100. Higher plDDT values correspond to higher confidence and vice-versa. When available, experimental data derived from biochemical and biophysical characterisation were also used to evaluate the 3D models.

**Figure 2:**
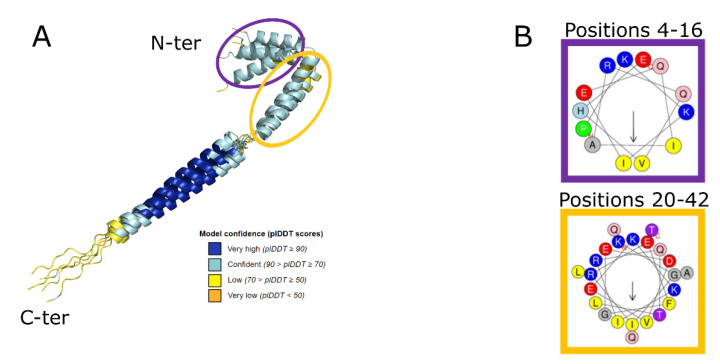
Modelling of UbiK monomer. (**A**) Structural alignment of 5 UbiK models predicted by AF2. Structural models were superimposed using Pymol. 3D models are displayed in cartoon coloured as a function of plDDT value. (**B**) Prediction of amphipathic helix profiles using HeliQuest server [26]. Hydrophobic and polar residues correspond to yellow and red/blue circles, respectively. Residue numbering corresponds to the Uniprot sequence (Uniprot ID: Q46868).

Analysis of the five models of UbiK (Figure 2A) showed a ranking of the global plDDT score between 75.3 and 80.9, indicating a good overall confidence of all 3D models. As described in the literature [25], the regions having low plDDT score values (coloured in yellow on Figure 2A) correspond to flexible regions such as the C-ter region or the loops between helices, whereas more structured regions such as helices (coloured in cyan and blue on Figure 2A) present higher plDDT scores ranging from 79.7 to 84.9. A comparison of the five 3D models revealed that the main differences reside in the orientation of these helices, without any other significant conformational change. Therefore, all 3D models can be considered equivalent, and the best prediction (highest mean plDDT score) was thus selected as reference. It is worth noting that the uncertainty on the N-ter (coloured in yellow and cyan in Figure 2A) could be explained by the conformational ambiguity of this region, already reported in the literature for BMFP family [13]. Indeed, the N-ter of BMFP proteins has been found to adopt several conformations depending on the environment. For example, this region can be disordered in the cytosol but structured as an amphipathic helix when interacting to the membrane. Interestingly, the N-ter helical region of UbiK was submitted to the HeliQuest server [26] (Figure 2B) which predicted a high hydrophobic moment of the two helices, with a higher value of the short one highlighted in purple (Figure 2A,B). These results indicate that these helices can be considered as amphipathic, in agreement with the literature related to the BMFP family [13]. Of note, we decided in the current study to use the helical conformation predicted by AF2 for subsequent modelling and analyses, without exploring further the conformation of this region.

**Figure 3:**
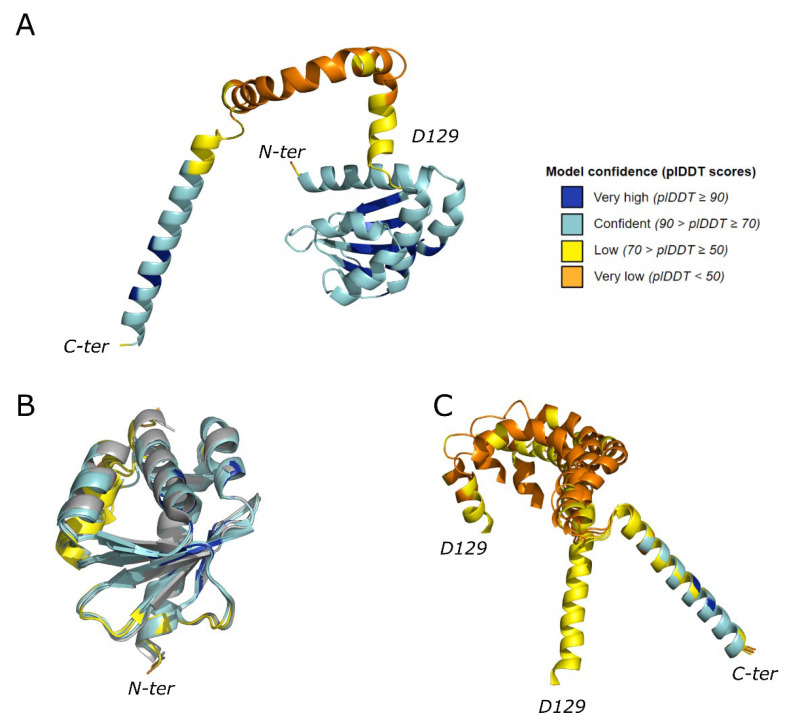
Modelling of UbiJ monomer. (**A**) Best AF2 model of the whole UbiJ represented as a cartoon and is coloured as a function of the pLDDT value. (**B**) Structural comparison of the 5 models of UbiJ SCP2 domain predicted by AF2 and crystallographic structure. The proteins are represented as a coloured cartoon, the X-ray structure in grey (PDB code: 6H6O) and the AF2 models coloured as a function of the pLDDT value. (**C**) Structural differences of the C-ter region of UbiJ (residues 129–201) observed in AF2 models. The structural alignments were performed on the C-ter helices (residues 171–201). The residue numbering corresponds to the Uniprot sequence (Uniprot ID: P0ADP7).

The first analysis to evaluate UbiJ models (best ranked shown in Figure 3A) was to compare the predicted SCP2 domain with the available X-ray crystallographic structures (PDB codes: 6H6O, 6H6N, 6H6P) [10]. Figure 3B illustrates one example of structural alignment between the five predicted 3D models and a crystallographic structure (PDB code: 6H6O). Structural superimposition between 3D models and X-ray structures, using the TM-align webserver (https://zhanggroup.org/TM-align/, accessed on 7 June 2022) [27], provided RMSD values ranging from 0.58 to 1.50 Å. As expected, these results indicate that the global fold of the SCP2 domain is modelled with a good confidence, confirmed by high values of pLDDT (72.9–82.0). However, the quality of the complete 3D models decreased when including the C-ter region, with global pLDDT scores varying between 65.2 and 73.9. This loss is mostly due to the low confidence of the region encompassing residues 129–165, which are likely to be flexible. Indeed, Figure 3C displays several orientations of this region (residues 129–162) after structural superimposition of the C-terminal helix (residues 171–201).

This first part of our work was focused on modelling of the monomeric proteins, and it sets the foundations for the modelling of the UbiJ-UbiK_2_ heterotrimer using AF2. Indeed, results obtained on the modelling of monomers are in agreement with the existing knowledge and experimental data, such as SCP2 in comparison with X-ray structures and helical C-ter regions for UbiJ and UbiK which were determined by circular dichroism [12,13,14]. Interestingly, we also identified a potential key flexible region in UbiJ (residues 129–165) which could assist SCP2 domain for its functional role in the binding and the release of UQ. Overall, the predicted 3D models show a good confidence, allowing us to pursue the UbiJ-UbiK_2_ heterotrimer modelling with the same approach.

### 2.2. UbiJ-UbiK_2_ Heterotrimer Modelling

To better understand and characterise the molecular interactions between UbiJ and UbiK, we proceeded to the modelling of the UbiJ-UbiK_2_ heterotrimer using AlphaFold2-multimer, a version of AF2 trained on oligomers. Based on earlier reports [10,12] suggesting a stoichiometry of two UbiK subunits for one UbiJ subunit in the complex, we built 3D models of the UbiJ-UbiK_2_ heterotrimer. By default, 25 structural models were generated by AF2-multimer using five different seeds to enhance conformational diversity. As expected, the assessment of the multimer quality is less trivial compared to the prior analyses on monomers. In the AF2 protocol, the confidence score corresponds to the combination of the pTM score used for monomers and the ipTM score for the interface. As described in the literature, the ipTM score is correlated to the dockQ metric [22], indicating that a protein interface can be well predicted when iPTM score is higher than 0.6. The twilight zone for a good confidence of the predicted interface is found between 0.5 and 0.6.

**Figure 4:**
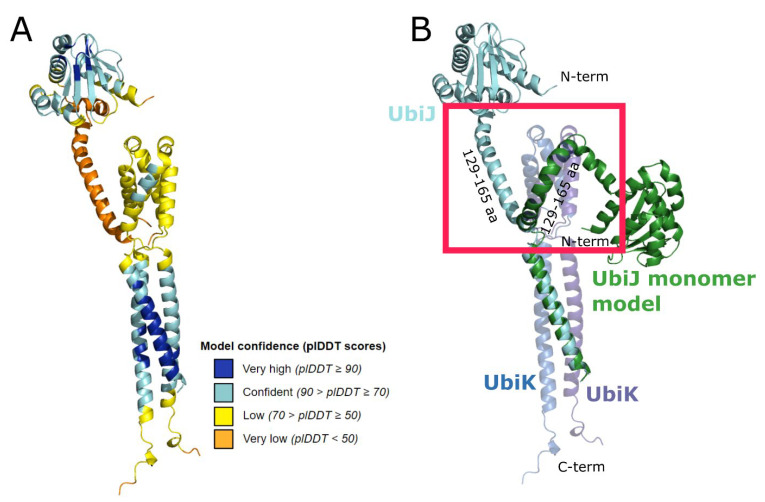
Modelling of the heterotrimer (UbiJ-UbiK_2_). (**A**) Heterotrimer prediction using AF2 coloured according to the plDDT score and shown in cartoon. (**B**) Structural superimposition of the best AF2 model and the UbiJ monomer, shown in green cartoon.

Analysis of the heterotrimeric modelling by AF2 revealed confidence scores (Ptm + iPTM) between 0.41 and 0.50. As shown in Figure 4A, the highest confidence regions correspond to the helices forming a coiled-coil, and the SCP2 domain which shows again a 3D fold highly similar to that observed in X-ray structures (RMSD < 1Å). The low confidence regions were found in the N-ter of UbiK (yellow-coloured helices), and the kinked helix of UbiJ corresponding to residues 121–170 (in orange). Interestingly, these regions were already identified to be of low confidence in analyses of the monomer models. When superimposing the monomer of UbiJ on the predicted heterotrimeric model (Figure 4B), it can be observed that the main structural difference comes from the orientation of the hinge region (residues 121–170) of UbiJ. This latter needs to undergo conformational rearrangements to avoid steric clashes with N-ter region of UbiK (red square in Figure 4B). Moreover, the conformational changes of the hinge region (residues 121–170) drastically affect the orientation of SCP2, which points in the opposite direction compared to the monomeric form. Finally, this result highlights that AF2 performs flexible-like docking by considering the environment in the multimer version.

**Figure 5:**
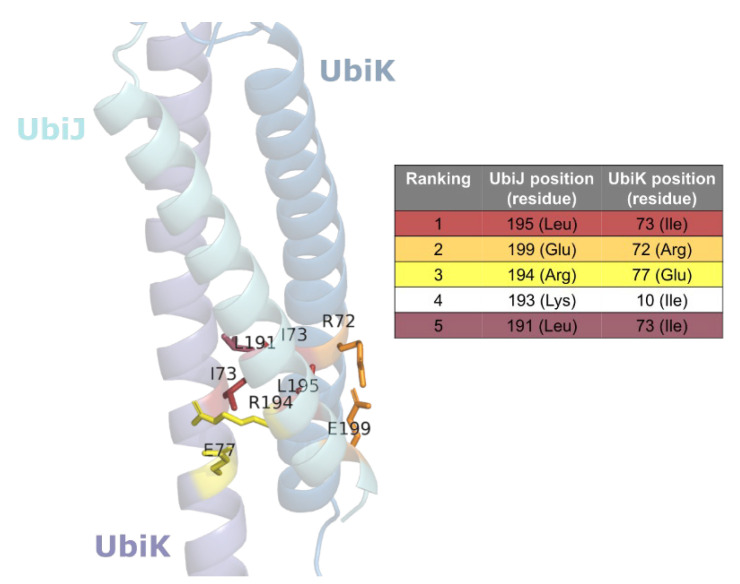
Co-evolution analysis. The top 5 best co-evolved residue pairs are listed in the table on the right and mapped onto the 3D structure of the heterotrimer on the left.

In spite of a better confidence on the interface described by a coiled-coil interaction, the iPTM values ranging between 0.43 and 0.53, indicate that further analyses are needed to validate the predicted interface. Thus, two additional sequence-based analyses were performed corresponding to coiled-coil prediction and co-evolution-based inter-residue contact prediction. First, the coiled-coil signature was predicted using the Deepcoil2 webserver (https://toolkit.tuebingen.mpg.de/tools/deepcoil2, accessed on 4 July 2022) [28,29]. The results of this analysis are shown in Figure S1A for UbiJ and in Figure S1B for UbiK. Interestingly, for both proteins, the C-ter regions were identified as potentially forming coiled-coil motifs, with however a lower probability for UbiJ. Moreover, the highlighted residues (black boxes in Figure S1), corresponding to residues 176–196 for UbiJ and 53–80 for UbiK, are in agreement with the AF2 prediction and the literature reports [13,14]. Co-evolution analysis approaches have already been reported to be of great interest to predict inter-residue contacts with good accuracy [19] and they have been integrated, amongst other features, in AF2. Therefore, we performed a co-evolution analysis using the I-COMS server. For that, we used a sequence dataset that was specifically prepared for each protein according to their corresponding protein family. Indeed, only sequences from related organisms (gamma- and beta-proteobacteria) showing a similar composition in ubiquinone biosynthetic proteins to the one observed in *E. coli* and thus potentially having the same protein organisation for the Ubi metabolon (in this case three hydroxylases and two methyltransferases) were selected for the dataset construction. Altogether, the final dataset contains 1336 sequences of UbiJ and UbiK that have been aligned in a concatenated multiple sequence alignment (MSA). Co-evolved residues were predicted using the plmDCA algorithm, which is the one used by CCMpred [30] and considered so far as being the best statistical method for contact prediction [20]. It is worth noting that this type of analysis is more efficient when using a non-redundant dataset. For this reason, a sequence identity cut-off of 62% was used to cluster the concatenated UbiJ and UbiK sequences, which resulted in 282 clusters. Only the 100 best scores were plotted and analysed to find the optimal number of best residue pairs (Figure S2). When inspecting the plmDCA score distribution, two cut-off points can be highlighted. The first one is viewed between the third and the fourth best predictions, with a score of 0.198 and 0.151, respectively (red lines in Figure S2). The second one is observed after the fifth best prediction with a score of 0.125, where the distribution starts decreasing slowly to reach an asymptote around 0.1 (see blue lines in Figure S2). For this reason, i.e., no significant change in score after top 5, this last cut-off point (top 5) was chosen to select the best predictions. These best residue pairs are listed in Table 1 and mapped on the best 3D model predicted using AF2 (Figure 5). A distance analysis was performed to determine if the selected pairs were in contact, using a distance threshold of 5 Å between two heavy atoms from two different protein chains [31]. Interestingly, out of the five residue pairs, four were found in contact in the AF2 heterotrimer best model (Table 1). Due to the stoichiometry, i.e., one UbiJ for two UbiK chains, the residue contacts are observed for only one UbiK chain (A or B) as the residue pairs ranked 2 and 3 or for both chains as shown for the best residue pair as the residue pair ranked 1 and 5.

Two types of interactions are observed. First, van der Waals, or hydrophobic, interactions involving I73 (UbiK) with L195 and L191 (UbiJ) are found, with I73 residue appearing determinant in the coiled-coil interactions. Second, electrostatic interactions (salt bridges) involving E199 (UbiJ) with R72 (UbiK) and R194 (UbiJ) with E77 (UbiK) are observed. These interactions are consistent with the ones found usually in Protein–Protein interactions [32], such as coiled-coil interactions [15,16,33]. The only exception is found for the fourth best pair between K193 (UbiJ) and I10 (UbiK), which shows high distance values incompatible with contacts/interactions (Table 1 and Figure 5), likely resulting from artefacts of the methods as already described in the literature [20].

Altogether these results suggest that the 3D models of the heterotrimeric complex predicted by AF2 are consistent with the coiled-coil interaction determined experimentally by Loiseau et al. [12] and provide a molecular insight of this co-evolved interaction. The best ranked 3D model of the UbiJ-UbiK_2_ complex was thus chosen for further analyses. Of importance, a conserved interface for the other AF2 heterotrimer 3D models was observed. In order to further evaluate the stability over time of the complex and more particularly of the overall interface and the co-evolved residue pairs, we subjected the system to MD simulations.

### 2.3. Heterotrimer Stability Inferred by MD Simulations

**Figure 6:**
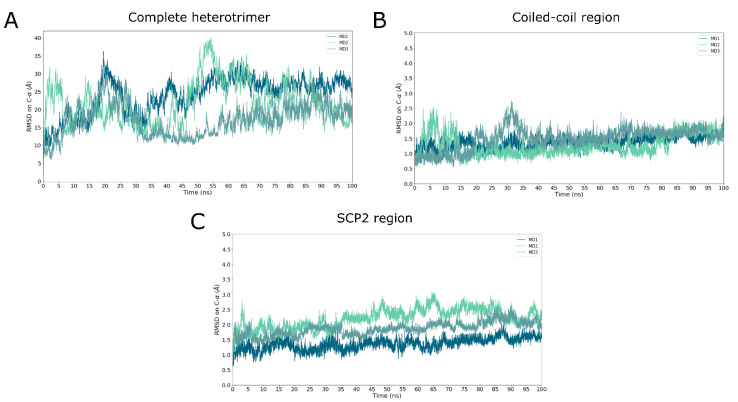
Heterotrimer stability in solution using all-atom MD simulation. Cα-RMSD is plotted as a function of MD time (ns) for the whole system (**A**), only for the coiled-coil region (**B**) and for SCP2 domain (**C**). For (**A**,**B**), the RMSD fit was done on Cα atoms of the coiled-coil region, which is defined by residues 171–201 for UbiJ and 49–85 for two chains of UbiK. For (**C**), the RMSD fit was done on SCP2 domain, defined by residues 1–121 of UbiJ. For all RMSD calculations, the first frame of each MD production was used as reference for RMSD calculations.

In order to investigate the stability of the best heterotrimer model, three all-atom MD simulation of 100 ns were first performed in solution. The root mean squared deviation (RMSD) based on Cα atoms was monitored and plotted as a function of the simulation time for the whole system and only for the coiled-coil region (Figure 6A,B). Of note, the RMSD fit was performed on Cα atoms of the coiled-coil regions defined by residues 171–201 for UbiJ and 49–85 for the two chains of UbiK. This strategy was chosen because the coiled-coil is considered to be the core region. For all MD simulations, the results show that the coiled-coil is very stable with an average RMSD of around 1.4 Å ± 0.3, whereas RMSD for the whole system reaches values higher than 10 Å. To better understand the contribution of these high values, RMSD profile based on Cα atoms for only SCP2 was monitored and is shown in Figure 6C. It shows that SCP2 is also stable with average RMSD value around 2.0 Å ± 0.3.

To further investigate the coiled-coil stability, the predicted contacts from the top 5 of co-evolved residue pairs (Table 1) were monitored along the MD trajectory (Figure S3). The distance profile shows low variations, indicating that the interactions are maintained throughout the simulation. Moreover, two clusters containing leucine residues were identified suggesting contacts such as “Leucine Zipper”. These residue contacts are known to be key in maintaining the coiled-coil region. Their stability was analysed by monitoring the minimal distances between residue pairs. The results are displayed in Figure S4 and show that these contacts are also stable with an average of around 5 Å.

Altogether, these results indicate that the coiled-coil and the SCP2 domain are stable, emphasising the prediction accuracy for these regions. As expected, conformational rearrangements contributing to high RMSD values for all MD simulations (Figure 6A) belong to the hinge regions such as the disordered or flexible C-termini or the region defined by residues 129–165 for UbiJ. Indeed, this latter has already been highlighted in the modelling sections and its flexibility is visually exemplified in snapshots taken at 0, 20 and 70 ns in Figure S5.

**Figure 7:**
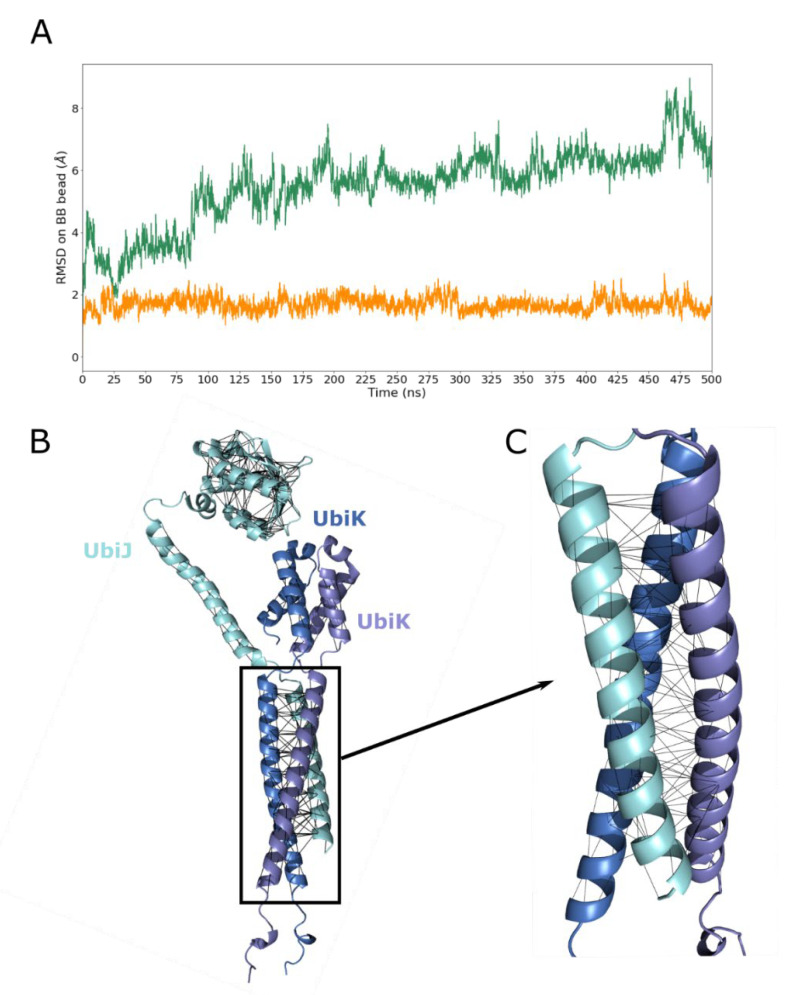
Heterotrimer stability in solution using CG MD simulation. (**A**) RMSD based on the CG backbone beads (BB) is plotted as a function of MD time (ns) for only intra-chain Go-like model (green) and adding inter-chain Go-like model (orange). The RMSD fit was done on BB beads of the coiled-coil region, which is defined by residues 171–201 for UbiJ and 49–85 for two chains of UbiK. The first frame of the MD production was used as reference for RMSD calculations RMSD on the backbone beads on 500 ns of AF2 model with and without Gō-like model (**B**) AlphaFold2 heterotrimer shown in cartoon with the used Gō-like model in black lines. (**C**) Enlarged view of the Gō-like model on the coiled-coil region.

To enhance the sampling of these flexible regions, coarse-grained (CG) MD simulations were performed in solution, using the recent Martini 3 force field. The coiled-coil stability observed in all-atom simulations was used as a reference to validate CG simulations. As described in the literature [34], Go-like constraints enable to improve the dynamics and the sampling of CG simulations. For this reason, CG MD simulations were first performed using Go-like models for intra-chain residue contacts (see green curve in Figure 7A). As shown, when no Go-like constraint is applied between protein chains, RMSD values of the coiled-coil region increase significantly reaching values around 7 Å at the end of MD, and with an average RMSD of 5.5 Å ± 1.2 over time. This result means that the coiled-coil is unstable and thus, Go-like model of inter-chains (Figure 7B,C) was also applied to reach a similar profile to the one obtained in all-atom simulations. This strategy is recommended by Martini developers to avoid residue exclusion. It shows that the coiled-coil RMSD profile is comparable to the one of all-atom, with an average of 1.4 Å ± 0.2 and 1.7 Å ± 0.2, for all-atom and CG representations, respectively.

The fairly good agreement between all-atom and CG MD simulations in water provided encouraging grounds for subsequent studies including a membrane environment. Indeed, one part of our study aimed at better characterising the potential interactions between the heterotrimer (UbiJ-UbiK_2_) and the lipid bilayer membrane. Therefore, CG MD simulations were needed to decrease the system size and to enable better conformational sampling of the complex molecular system.

### 2.4. Heterotrimer-Membrane Interactions

In this section, the main objective is to better understand the interactions between the heterotrimer UbiJ-UbiK_2_ and the lipid bilayer membrane. A multi-scale MD simulation approach, i.e., a series of CG and all-atom MD simulations [35], was used to tackle this question. For both types of MD simulations, the lipid bilayer membrane was described as realistically as possible with a composition of POPE:70, POPG:25, LNDCL2:5. This ratio corresponds to the one of the inner membranes of Gram-negative bacteria [36], which is consistent with the organism of this study, *E. coli*.

#### 2.4.1. Adsorption of UbiJ SCP2 on the Membrane

CG MD simulation of 1 µs was first performed with UbiJ SCP2 as a single protein, in order to investigate its adsorption on the membrane. Indeed, based on the biological role of SCP2 domain, we assumed that single UbiJ SCP2 could interact with the membrane. This strategy was carried out to reduce the complexity of the system and to enhance the sampling which allowed us to have a first insight of the UbiJ SCP2 binding. To allow the diffusion of the SCP2 before adsorption, this latter was placed at a minimal distance of 21 Å between the protein and the membrane, which corresponds to about two times the cut-off for non-bonded interactions. The diffusion and the adsorption can be analysed through the monitoring of the distance between the centre of mass of the SCP2 domain and the polar head groups (Figure S6).

**Figure 8:**
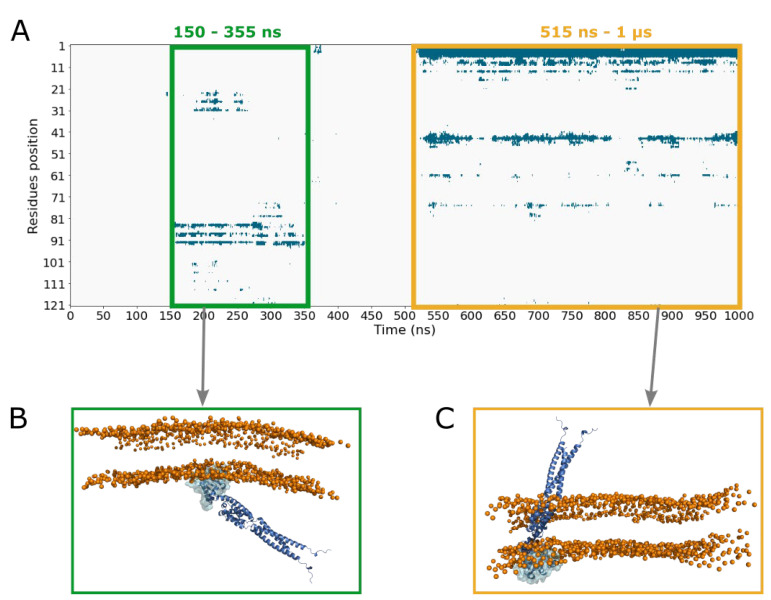
Adsorption of the single SCP2 domain onto the membrane from CG simulation. (**A**) Contact map of the SCP2 domain with the membrane in CG simulations, (**B**) representative snapshot of the first identified binding mode according to contact map (around 200 ns), (**C**) representative snapshot of the second identified binding mode according to contact map (around 880 ns). For both snapshots, single SCP2 domain adsorbed on the membrane is shown in transparent surface. The full heterotrimer is displayed in cartoon representation and was superimposed onto the SCP2 domain. For clarity, orange spheres represent the polar head groups of the membrane.

**Figure 9:**
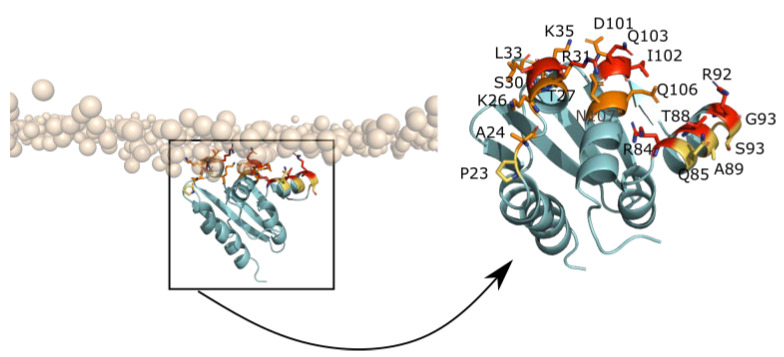
Key residues of UbiJ SCP2 domain in contact with the membrane. On the left, the UbiJ SCP2 domain is shown in cartoon representation and key residues in contact with the membrane in sticks. For clarity, only polar head groups of the membrane are shown in transparent orange spheres. A zoom of only SCP2 domain is shown on the right, with a colour code for residues in contact with the membrane as follows: red for common residues, orange and yellow for specific residues from all-atom and CG simulation, respectively.

In order to investigate the residues involved in the adsorption, a contact map between residues and the membrane as function of time was computed and is plotted in Figure 8A. In this analysis, a contact is defined between one protein bead and membrane bead within a threshold of 5 Å. Figure 8 shows that UbiJ SCP2 domain is able to establish some interactions with the membrane after diffusion in water. We can observe two types of binding sites of the protein on the membrane. The first one is viewed between 150 and 355 ns and the second one is observed between 515 ns and 1 µs. To discriminate the most probable binding mode, the full heterotrimer was superimposed onto the SCP2 domain and the resulting structural superimposition is displayed in Figure 8B,C using one representative snapshot per binding site. This analysis revealed clearly that the second adsorption mode is not compatible with the binding of the full heterotrimer, as the coiled-coil region is crossing the lipid membrane. For this reason, the first binding mode was selected for further analysis. The structure having the deeper anchoring shown in Figure S6 was considered as the most energetically favourable and so was chosen as the most representative snapshot. To verify the stability of this latter, MD simulation of 100 ns was performed in all-atom after backmapping. This later step enables refinement of the backmapped structure and validation of the stability of contacts with the membrane highlighted in CG representation. The contact profiles of CG and all-atom representations (Figure S7A,B) are similar with about 60% of native contacts remaining stable. The native contacts are selected from the CG conformation before backmapping, and the stability is defined when contacts occur more than 75% of the MD time. Amongst stable residues in contact with the membrane, 7 residues (S30, R31, R84, T88, R92, I102 and Q103) are common, 8 residues (A24, K26, T27, L33, K35, D101, Q106, N107) are specific to all-atom MD simulations and 5 residues (P23, Q85, A89, S93, G94) are specific to CG MD simulations. The listed residues are displayed in sticks in Figure 9 and are coloured as red for common residues, as orange for all-atom specific residues and as yellow for CG specific residues.

This result highlights the interest to refine coarse-grained models in order to increase the accuracy of the interactions. Indeed, positive charged residues such as K26 and K35 were found only in all-atom representations, which is consistent with the negatively charged membrane.

All these results suggest that this predicted binding mode of UbiJ SCP2 (see Figure 8B for CG representation and Figure 9 for all-atom representation) is biologically relevant and thus, it was further used for two additional analyses: (i) the adsorption stability of the heterotrimer, (ii) the release of ubiquinone.

#### 2.4.2. Adsorption of Full Heterotrimer on the Membrane

**Figure 10:**
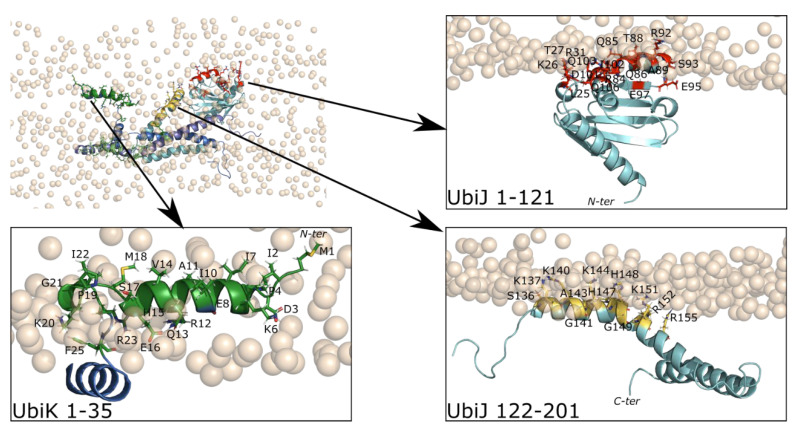
Heterotrimer model in interaction with the membrane. On the top left a top view of the adsorption is illustrated with the heterotrimer in cartoon and the polar head groups of lipid membrane in orange spheres. For clarity, three zoomed views are shown for key interacting regions. The stable residues in contact with the membrane are listed on the bottom. The colour code is the following: red for SCP2 of UbiJ, yellow for the residues 122–201 of UbiJ, green for residues of UbiK. Bold red labels correspond to common residues between single SCP2 and heterotrimer. Italic red labels correspond to specific residues from single SCP2 MD simulations.

The adsorption stability of the heterotrimer was investigated in two subsequent steps, first CG and then all-atom MD simulations. The starting point for CG representation corresponds to the most-anchored conformation of UbiJ SCP2 (Figure 8B). The AF2 heterotrimer in CG representation was aligned onto the SCP2 domain and then, a CG MD simulation of this system was run for 500 ns. As previously, this strategy allows us to enhance the sampling and to get a first insight of the molecular interactions for the full heterotrimer. The most anchored CG pose was then selected and backmapped to run MD simulation in all-atom. To analyse the adsorption, contact map as a function of MD time is plotted and is displayed for CG and all-atom representations in Figure S7C and 6D, respectively. The overall contact profiles appear similar in both representations, mostly in the SCP2 region of UbiJ and in the N-ter of UbiK. This result emphasises the stability of the heterotrimer adsorption onto the membrane. To validate the interface at the molecular level, analysis of stable contacts was performed as shown in Figure 9. Key amino acid residues in contact with the membrane were identified for the heterotrimer in all-atom representation and compared to previous residues listed in Figure 9 for the SCP2 region (Figure 10). Concerning the SCP2 region, 7 residues (L25, Q85, Q86, A89, S93, E95, E97) were specific to the heterotrimer simulation, 5 residues (A24, S30, L33, K35, N107) were specific to the single SCP2 analysis and 10 residues (K26, T27, R31, R84, T88, R92, D101, I102, Q103, Q106) were common (Figure 10).

Amongst the common residues, we can notice that 4 residues are positively charged (K or R) ensuring strong interactions with negative charges from the membrane lipid head groups. The emergence of specific contacts could be explained by the difference of the environment when modelling the full heterotrimer. Indeed, in the full system UbiJ-UbiK_2_, the SCP2 domain is not the only region interacting with the membrane. Regarding the rest of the UbiJ protein, the flexible region (129–165) previously discussed is found to establish stable contacts via 12 residues (S136, K137, R140, G141, A143, K144, H147, H148, G149, K151, R152, R155) shown in yellow sticks in Figure 10. Half of them are positively charged. Inspection of the interactions between UbiK and the membrane revealed 22 residues (M1-P4, K6-E8, I10-R12, Q13, H15-R23, F25, R61) in contact, which are shown in green sticks in Figure 10. Interestingly, all but one residue are located on the N-ter region, confirming the amphipathic property as illustrated in Figure 2B and in agreement with their role in the BMFP family. The only exception for UbiK is for the residue R61, which is located on the C-ter region and is still consistent with the negative charge of the membrane.

Together, these results illustrate that the interaction of the heterotrimer with the membrane is mediated mostly by two amphipathic helices of the N-ter region of UbiK and by the SCP2 and the flexible region (129–165) of UbiJ. The C-ter region of UbiK can interact weakly with the membrane. It should however be noted that consideration of the entire Ubi metabolon in the modelling could alter observed interactions between the membrane and the C-ter of UbiK since this part of the heterotrimer is likely to be engaged in interactions with other Ubi proteins of the metabolon.

### 2.5. Ubiquinone Release

In this section, we aim at exploring further the hypothetical role of UbiJ SCP2, which is the binding of UQ_8_ biosynthetic precursors and the release of UQ_8_ into the membrane. Our results have shown that the interface region between SCP2 and the membrane is consistent when considering only the single SCP2 or the full heterotrimer. From this point, it was decided to study in detail the binding and release of UQ_8_ from the single UbiJ SCP2 domain.

**Figure 11:**
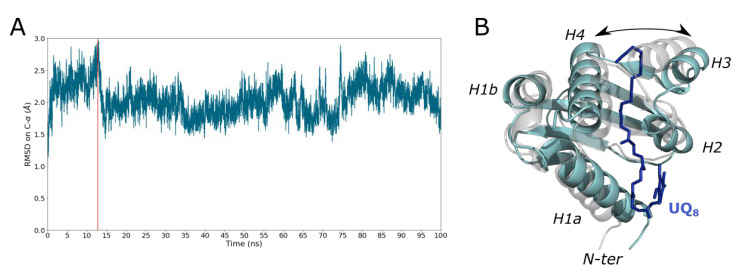
Binding of UQ_8_ with the UbiJ SCP2 using all-atom MD simulation in water. (**A**) RMSD based on Cα atoms is plotted as a function of MD time, with X-ray structure (PDB code: 6H6O) as reference. (**B**) The snapshot selected for docking analysis. SCP2 is shown in cartoon representation (grey for the initial and cyan for the selection highlighted by a vertical red line on the RMSD plot) and UQ_8_ in blue sticks. Key helices of the SCP2 domain are labelled H1a, H1b, H2, H3 and H4. The double-headed arrow highlights the increase of the distance between H3 and H4 to accommodate the hydrophobic tail of UQ_8_.

To predict the UQ_8_ binding into SCP2, docking experiment was first carried out using Autodock4 and the X-ray structure (PDB code: 6H6O) as protein reference. Unfortunately, the docking results were not satisfying, due to the closed conformation of the crystallographic structure. To tackle this issue, a manual docking of the UQ_8_ was guided through structural comparison of SCP2 X-ray structures in complex with lipid (PDB code: 4jgx, 1pz4) and pocket search. This manual docking led to positioning the UQ_8_ into a highly hydrophobic cavity, which is in adequation with the tail of UQ (Figure S8A). To check the stability of the SCP2-UQ_8_ complex, a 100 ns MD simulation was performed in water. The RMSD based on Cα-atom revealed that the SCP2 bound to UQ_8_ remains stable throughout the simulation with an average value of 2.0 Å ± 0.3 (Figure 11). During all the trajectory, UQ_8_ is stable and remains in the groove defined by the β-sheets and the helices (H2, H3 and H4) as shown in Figure 11. To validate this binding pose, the most open conformation was selected based on the RMSD profile in Figure 11 and used for a new run of docking. Interestingly, the lowest energy docking pose matched the one from the snapshot (Figure S8B). This result reinforces the confidence on the binding pose, especially the orientation of the quinone head which seems to be more consistent with respect to the potential role of SCP2.

Therefore, this binding pose was used as reference to place UQ_8_ into the most anchored SCP2 structure described in Figure 8B. In order to accommodate UQ_8_ into the adsorbed SCP2, a MD simulation of 100 ns in all-atom was performed before running free energy calculations.

To explore the energetics driving the release of UQ_8_ towards the lipid membrane, a two-step strategy was used: (i) Pulling protocol to find the release pathway and generate conformations; (ii) umbrella sampling (US) coupled with the weighted histogram analysis (WHAM) method to compute the free energy profile of the release (see subsection *Umbrella Sampling* in Material and Methods). To find the shortest path for the release of UQ_8_, the hydrophobic tail of UQ_8_ was pulled from UbiJ SCP2 towards the membrane (Figure S9A). Interestingly, the pulling force profile shows two energy barriers to surpass (Figure S9B). The first barrier is observed at 50 ps with a pulling force of 400 kJ/mol/nm and seems to correspond to conformational changes of SCP2 to release UQ_8_. The second barrier is viewed at 300 ps with a pulling force of 800 kJ/mol/nm, and seems to be more related to the crossing of the polar head groups.

**Figure 12:**
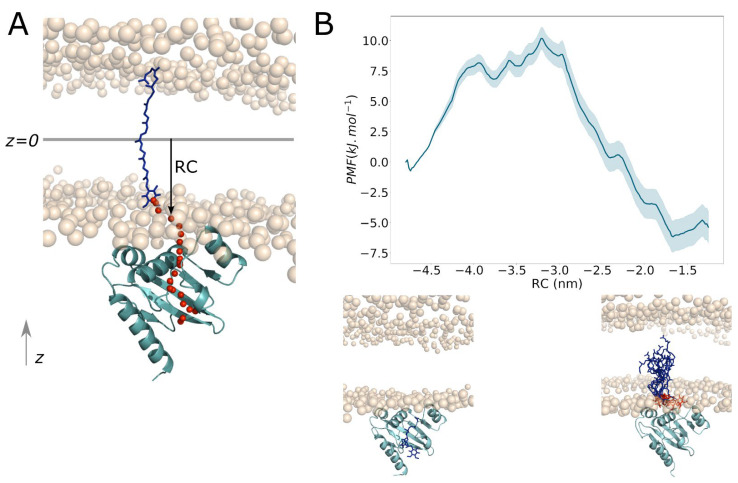
Free energy calculations of UQ_8_ release. (**A**) A schematic representation of the release pathway is displayed. The SCP2 domain is shown in cyan cartoon whereas the reference points used as reaction coordinates RC are shown in red spheres (mass centres of the quinone head). The red spheres are uniformly spaced of 2 Å along the z-axis. UQ_8_ is represented as blue sticks at the end of the pulling. The orange spheres correspond to polar heads of the membrane. (**B**) PMF of the UQ_8_ release along the reaction coordinate RC shown by a black arrow as an example in (**A**). Two snapshots illustrate the UQ_8_ position according to the two main basins.

Along this pulling pathway, 20 conformations (US windows) were chosen according to a uniform threshold of 2 Å along the z coordinate of centre of mass of the quinone head (red spheres in Figure 12A). Therefore, the reaction coordinate (RC) for US corresponds to the orthogonal vector between the centre of mass of quinone head and the middle plan of the lipid set to 0 as exemplified with an arrow in Figure 12A. It shows that only z position of the centre of mass of quinone head is constrained during US simulations. Constraining the quinone head of UQ_8_ during US simulations allows the sampling of the hydrophobic tail during the release, which is more biologically relevant. Thus, the potential of mean force (PMF), describing the energetics driving the UQ_8_ release, was computed along this reaction coordinate and is shown in Figure 12B. The PMF profile shows two minima, corresponding to UQ_8_ bound into the SCP2 domain (on left snapshot in Figure 12B, with a value of −0.7 kJ/mol) and to UQ_8_ inside the membrane (on the right in Figure 12B, with a value of −6.2 kJ/mol). It has to be noticed that the uncertainty is higher for the second basin, which could be explained by the sampling time required for the dynamics of the hydrophobic tail of UQ_8_ inside the membrane. However, the uncertainty does not seem to be significant and a good overlapping between US windows is observed (Figure S10A). Moreover, the evolution of the PMF profile as function of sampling time (Figure S10B) was investigated and the trend seems to be similar after 20 ns/window, indicating that calculations are converging. Therefore, all these data indicate a good confidence on the US profile. Thus, the free energy difference (ΔG) between two minima is about −5.5 kJ/mol ± 1.3 in favour of the UQ_8_ inside the membrane medium. The lowest minimum corresponds to the quinone head of UQ_8_ close to the polar head groups of the membrane, while the hydrophobic tail is free to interact with fatty acid chains of lipids. This preferential conformation was also shown by Galassi et al. through free energy calculations to study the insertion of the ubiquinone into a POPC bilayer [37]. Thus, the energy transition barrier to leave the SCP2 groove is about 11 KJ/mol (about 3 kcal/mol), with a peak of 10.2 kJ/mol at −3.21 nm (RC). Therefore, we think that this energy barrier could be overcome with some conformational changes of the SCP2 domain. Interestingly, this energy barrier is in the same order of magnitude than the one for the ubiquinone release from the respiratory complex I towards the membrane [38]. Moreover, we believe that this free energy barrier could have been lower if the entire complex had been modelled, which could help facilitate the release of UQ_8_, by applying some favourable forces to enable conformational changes.

**Figure 13:**
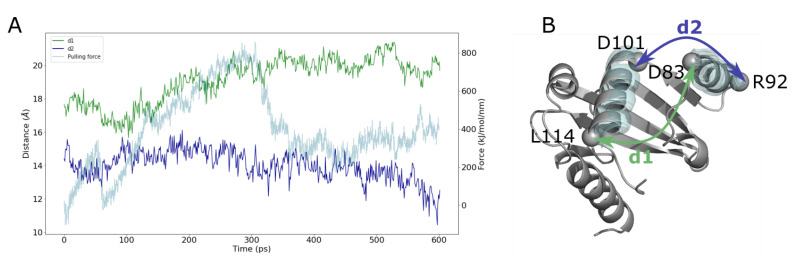
Main conformational change for UQ_8_ release. (**A**) Time evolution of the distance between residues D83 and L114 (d1, in green), and the distance between residues R92 and D101 (d2 in blue). Pulling force is plotted on y2-axis as a function of pulling time. (**B**) Mapping of d1 and d2 on the 3D structure of SCP2 domain shown in cartoon representation. The fluctuations of two key helices (H4 and H3) are shown in transparent cyan.

An interesting conformational change observed during the pulling is an opening between helices H3 and H4. This conformational change is correlated with a change of the distances between D83 and L114 (d1), and between R92 and D101 (d2) (Figure 13). The increase of the d1 distance is correlated to the decrease in d2 distance, which indicates an opening of the protein, that could facilitate the way out of the UQ_8_. The reason for this opening, which occurs mainly between 100 and 400 ns, corresponds to the higher force variations during the pulling (Figure S9B). These results suggest that a rearrangement of these helices (H3 and H4) is required to facilitate the release of UQ_8_ in the membrane.

## 3. Material and Methods

### 3.1. Co-Evolution Sequence Dataset

We downloaded one genome per species of beta- and gamma-proteobacteria from the NCBI database (last accessed October 2020) and obtained respectively 419 beta- and 1225 gamma-proteobacteria genomes. We then annotated these genomes for proteins of the ubiquinone biosynthetic pathway. To this end we used HMM (hidden Markov model) protein profiles that were built on a set of phylogenetically curated sequences [39]. A similarity search with the HMM protein profiles was run against the selected genomes using the hmmscan program from the HMMER suite (v3.3.2) [40]. Proteins with HMMER hits with an e-evalue < 0.001 and with a protein profile coverage >50% were selected. Finally, only the genomes showing a similar composition in ubiquinone biosynthetic proteins to the one seen in *Escherichia coli* (three hydroxylases and two methyltransferases), had their UbiJ and UbiK proteins extracted for analyses. For each UbiJ and UbiK dataset, multiple sequence alignment (MSA) was performed using MUSCLE [41] with BLOSUM62 as substitution matrix [42] and default parameters.

### 3.2. Co-Evolution Prediction

Prediction of co-evolved amino acid residues was performed using I-COMS [43] (Interprotein COrrelated Mutation Server) web server (http://i-coms.leloir.org.ar/index.php, accessed on 28 June 2022). Our precomputed multiple sequence alignment (MSA) (see Section 3.1) was used as input. plmDCA (CCMPred) algorithm [30] with default parameters (cluster) was chosen to predict the co-evolved pairs.

### 3.3. Molecular Modelling Setup

Local version of Alphafold2 from GitHub (https://github.com/deepmind/alphafold, accessed on 7 April 2022) was used with default parameters to model monomeric (Alphafold2 V2.1) and multimeric (Alphafold2-multimer v2.2) structures of UbiJ and UbiK.

Regarding monomer modelling, several MSA were built as follows: (i) JackHHMER (HMMER 3.3.2 [44]) and HHBlits (HHBlits 3.3.0 [45]) were used to search for sequences from Uniref90 (v2020_01 [46]), MGnify (v2018_12 [47]), Uniclust (v2018_12 [48]) and Big Fantastic Database (BFD). (ii) hmmsearch (HMMER 3.3.2) was used to search for sequences having structural information from the PDB70 database [49]. For each targeted sequence (UbiJ and UbiK), five models were generated.

Regarding multimer modelling, the only difference is the addition of UniProt database for sequence search and the generation of 25 models at the end.

For both strategies, all 3D models were relaxed with default parameters.

### 3.4. Molecular Dynamics Simulation Setup

All MD simulations were performed using Gromacs 2021.3 [50] with CHARMM36 [51] and Martini 3 [52] force-fields for atomistic and coarse-grained (CG) models, respectively.

For all systems with the membrane, the composition of lipids was the following: palmitoyloleoyl phosphatidylethanolamine (POPE, 70%), palmitoyloleoyl phosphatidylglycerol (POPG, 25%) and cardiolipin (LNDCL2, 5%). This ratio is in agreement with the literature [36]. Membrane bilayer was built using CHARMM-GUI [53] or insane script [54] for all-atom and CG systems, respectively.

#### 3.4.1. All-Atom MD Simulations

All atom MD simulations were run with CHARMM36 for protein and lipids, whereas TIP3 model was used to model explicit water molecules. A cut-off of 1.2 nm and a switch function from 1.0 to 1.2 nm were used for short-range electrostatic and van der Waals interactions, respectively. Long-range electrostatic interactions were treated with particle mesh Ewald (PME, parameters by default) and periodic boundary conditions (PBC). Covalent bonds involving hydrogen atoms were constrained using LINCS algorithm with default parameters. All systems were neutralised with a concentration of NaCl of 0.15 mM.

For MD simulations with the lipid membrane, CHARMM-GUI (https://www.charmm-gui.org/, accessed on 27 May 2022) [53] protocol was used, corresponding to a series of minimisation, 3 NVT and 3 NPT equilibrations with decreasing constraints. The production phase was run without constraint in NPT ensemble at 303 K and a timestep of 2 fs.

MD simulations in water were performed as follows, based on Gromacs tutorial [55]: (i) minimisation of the system with 50,000 steps of steepest descent algorithm, (ii) 100 ps of NVT equilibration, (iii) 100 ps of NPT equilibration, (iv) production phase without constraints and a timestep of 2 fs. During equilibration phases, constraints of 1000 KJ/mol on the protein backbone were set. MD simulations were run at 303 K.

#### 3.4.2. Coarse-Grained MD Simulations

All coarse-grained (CG) systems were prepared using Martinize2 protocol [56]. Go-like model with default parameters was applied to constraint the secondary structures close to the native protein fold. CG simulations were performed using Martini3 force field for protein, lipid and explicit water models. MD simulation setup was based on CHARMM-GUI protocol for Martini 2 with default parameters. Thus, a similar strategy as all-atom MD simulations was used. A minimisation and a series of NVT and NPT equilibration simulations were performed with a continuous release of the constraints, in the case of membrane systems.

Regarding MD simulations in water, a single NPT equilibration with constraints was performed, as suggested by Martini developers (http://cgmartini.nl/index.php/2021-martini-online-workshop/tutorials/564-2-proteins-basic-and-martinize-2, accessed on 31 May 2022). Whereas the production file was based on CHARMM-GUI protocol, using a cut-off of 1.1 nm for short-range interactions (van der Waals and electrostatics). PME algorithm for long-range electrostatic interactions was applied and the relative dielectric constant value was set to 15 as recommended. All CG systems were neutralised with a NaCl concentration of 0.15 mM.

CG2AT approach [57] was used for the whole systems to convert coarse-grained snapshot to all-atom one (backmapping).

### 3.5. Binding and Release of UQ_8_

#### 3.5.1. Ubiquinone Docking Setup

The structure of the ubiquinone was extracted from the UQ8 entry in the PDB. UQ_8_ was docked only onto the SCP2 domain of UbiJ using Autodock4 [58]. The protein was considered as rigid and Lamarkian Genetic Algorithm was used with 100 runs, a population size of 150 and other default parameters. The best docking pose was selected from the lowest energy and used as a reference for further analysis.

#### 3.5.2. Umbrella Sampling

Umbrella sampling (US) simulations were performed using Gromacs 2021.3. Conformations used for US simulations were generated through the pulling code of Gromacs software. The pulling was performed between the centre of mass of SCP2 and a carbon atom (C33) close to the end of UQ_8_ tail. The pulling vector was directed according to z-axis towards the membrane (Figure S9A), with a pulling rate of 0.01 nm/ps, a force constant of 1000 kJ/mol/nm and a number of steps of 600.

From the pulling trajectory, 20 windows uniformly spaced of 2 Å along the z coordinate of the mass centre of the quinone head were defined to perform US simulations. Unlike pulling, the reaction coordinate for US was based on the orthogonal vector defined by the middle plan of the membrane bilayer and the z-axis of the mass centre of the quinone head (Figure 12A). Each window was sampled for 25 ns, with same simulation parameters as described for classical MD simulations and with a force constant of 1000 kJ/mol/nm to constraint the reaction coordinate around the reference conformation. The sampling of US simulations was analysed and PMF was built using *gmx wham* tool from Gromacs [59]. To compute the errors on PMF, bootstrap analysis was carried out using *gmx wham*, with b-hist method and 100 repetitions.

## 4. Conclusions

In this study, we provide several new insights into the functional role of UbiJ and UbiK in UQ biosynthesis. We demonstrate that UbiJ-UbiK_2_ complex is stabilised by a coiled-coil interaction. Interestingly, this interaction was confirmed through co-evolution analysis, showing that this information could be used for studying similar proteins in other organisms. We also show that the heterotrimer interacts intimately with the membrane, suggesting the heterotrimer as the privileged entity to mediate the communication between the membrane and the Ubi metabolon. At the molecular level, regions such as the N-ter amphipathic helices of UbiK and the SCP2 domain of UbiJ were found to interact with the membrane. These results are in agreement with some previous works described in the literature. A list of key residues for the membrane interactions is given which could be tested experimentally by mutagenesis. Finally, our modelling and calculations show that a low energy barrier of about 3 kcal/mol is required to release UQ_8_ from the SCP2 domain of UbiJ, suggesting a possible pathway for newly synthesised UQ_8_ to leave the Ubi metabolon and reach its final destination, the cytoplasmic membrane. One resulting question pertains to the way the Ubi metabolon functions in vivo: are the enzymes of the Ubi metabolon always in contact with UbiJ-UbiK_2_, in which case the complex might be considered as a single entity constantly associated to the membrane, or does the UbiJ-UbiK_2_ heterotrimer form a docking platform allowing transient interaction with the Ubi metabolon when release of the UQ_8_ product into the membrane is needed? Additional experimental and in silico studies are definitely needed to elucidate the details of this fascinating Ubi metabolon.

## Figures and Tables

**Table 1:** Top 5 best co-evolved residue pairs. Distance columns correspond to the minimal distances between heavy atoms of UbiJ and UbiK. A and B indicate the first and the second UbiK chain. All distances are given in Å and residue numbering corresponds to the Uniprot sequence (Uniprot ID: Q46868 for UbiK and P0ADP7 for UbiJ).

Ranking	UbiJ Residue Position	UbiK Residue Position	Distance A (Å)	Distance B (Å)
1	195	73	4.25	3.39
2	199	72	11.08	2.79
3	194	77	2.72	11.59
4	193	10	46.13	43.07
5	191	73	3.53	5.66

## Data Availability

Not applicable.

## Supplementary Materials

The following supporting information can be downloaded at: https://www.mdpi.com/article/10.3390/ijms231810323/s1.
