# Supplementary material for: Towards Molecular Understanding of the Functional Role of UbiJ-UbiK2 Complex in Ubiquinone Biosynthesis by Multiscale Molecular Modelling Studies
Source: Int J Mol Sci. 2022 Sep 7;23(18):10323. doi: 10.3390/ijms231810323 (PMC9499169; doi:10.3390/ijms231810323)
Supplement: Supplementary file 1 [file ijms-23-10323-s001.zip › ijms-1866098-supplementary.pdf]

Supplementary Information

Towards molecular understanding of the functional role of UbiJ-UbiK2 complex in ubiquinone biosynthesis by multiscale molecular modelling studies

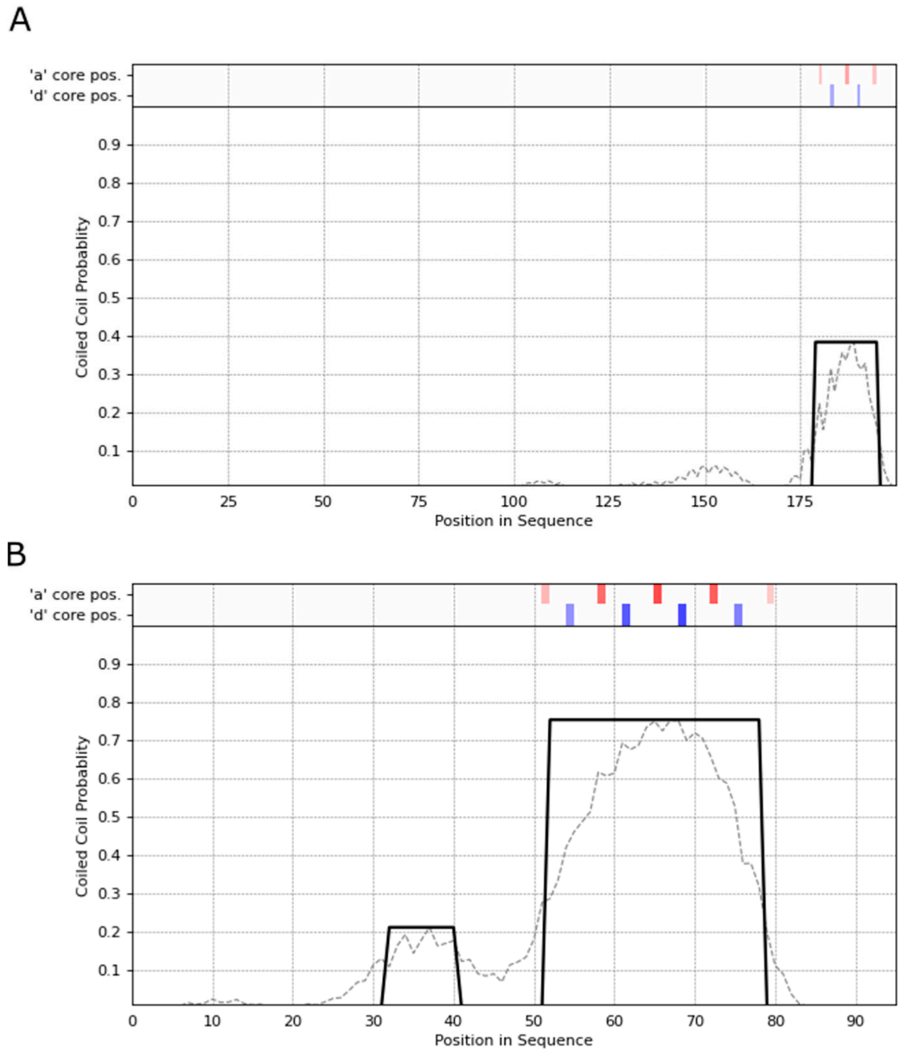

Figure S1. Prediction of coiled-coil region by DeepCoil2 of (A) UbiJ, and (B) UbiK. The coiled-coil probability is plotted as a function of the protein sequence. The black boxes highlight the predicted coiled-coil regions. The probability of a given residue to be in the interface core (core pos raw) is coloured according to a gradient of red or blue. Dark red or dark blue means a high probability and vice-versa.

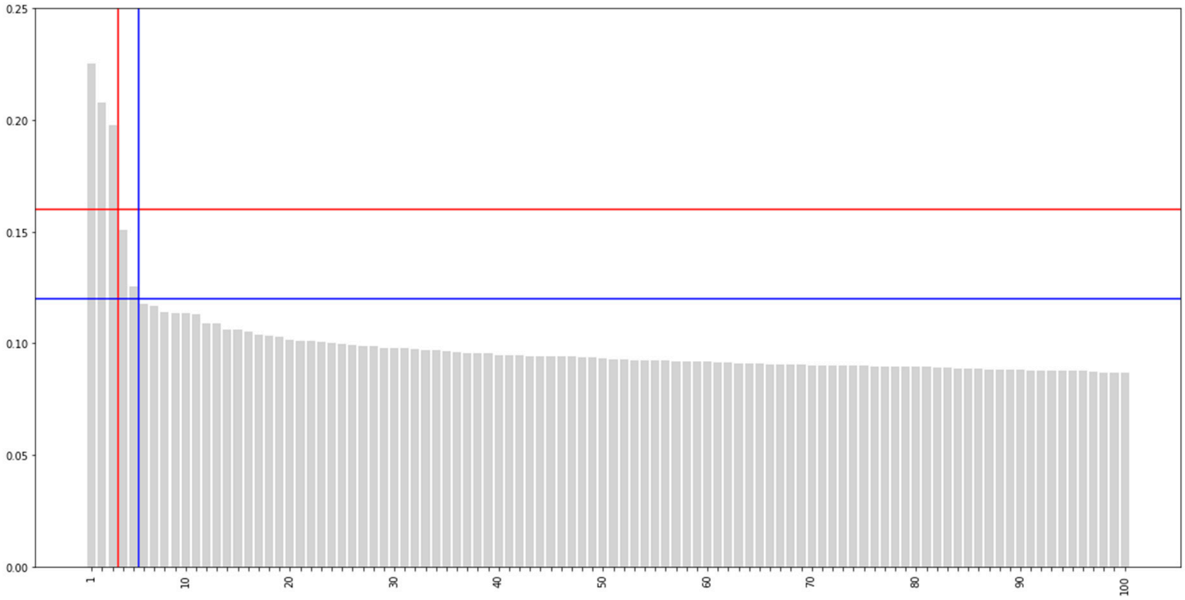

Figure S2. Plot of the 100 higher score with I-COMS. Two cut-off points are observed and highlighted by red and blue lines. The first cut-off is highlighted because it corresponds to a significant shift between the third and the fourth best score. The second corresponds to the beginning of the asymptote (no significant change between scores).

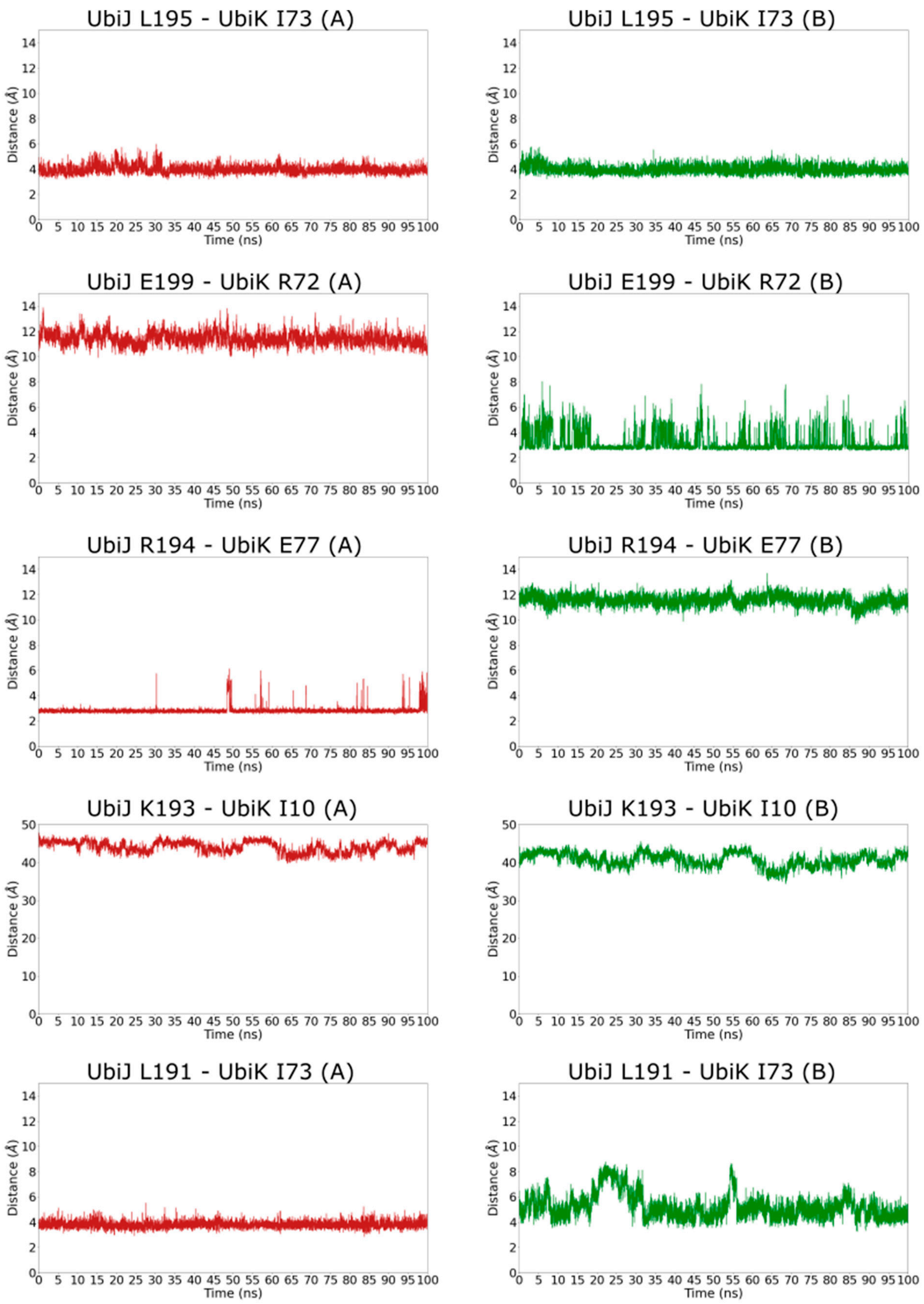

Figure S3. Stability of the co-evolved residues predicted by I-COMS. Minimal Distances between two heavy atoms are plotted as a function of the MD simulation time for the top 5 of coevolved residue pairs (labelled on the top of each plot). A and B correspond to the chain A and the chain B for UbiK.

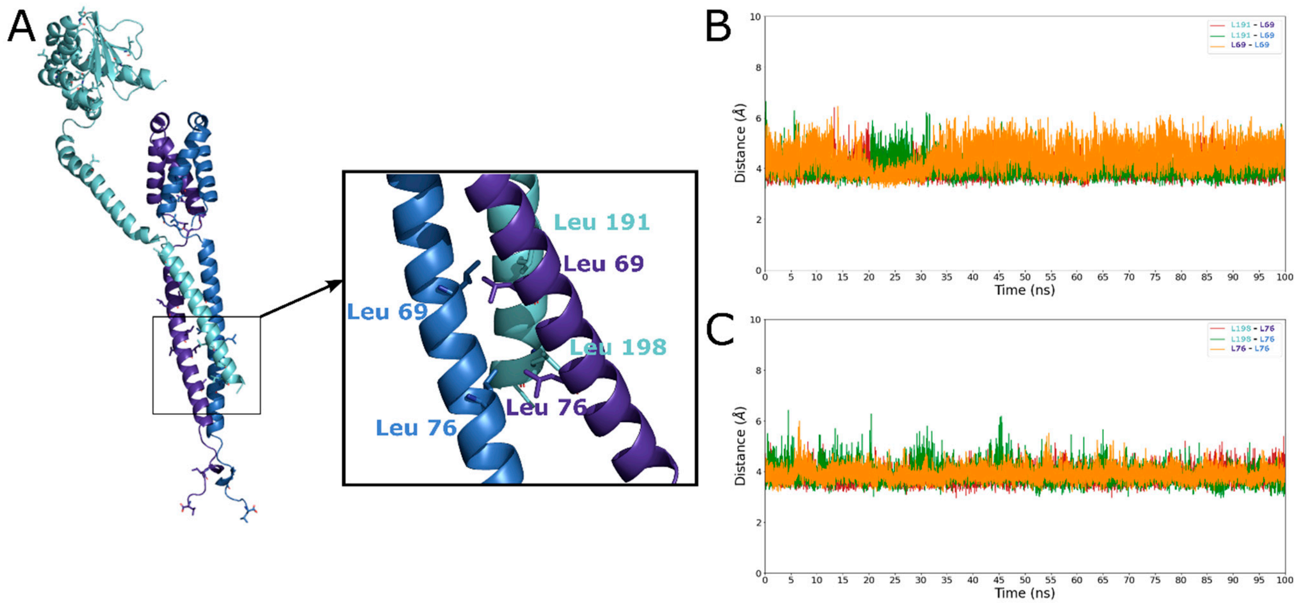

Figure S4. Stability of "Leucine Zipper". (A) Zoom on two clusters of Leucine residues in the coiled-coil (black square). The protein chains are shown in cartoon, whereas Leucine residues are displayed in sticks. The color code is the same as Figure 4B in the Main Text. (B) and (C) Minimal Distances between two heavy atoms are plotted as a function of the MD simulation time for Leucine pairs shown in (A).

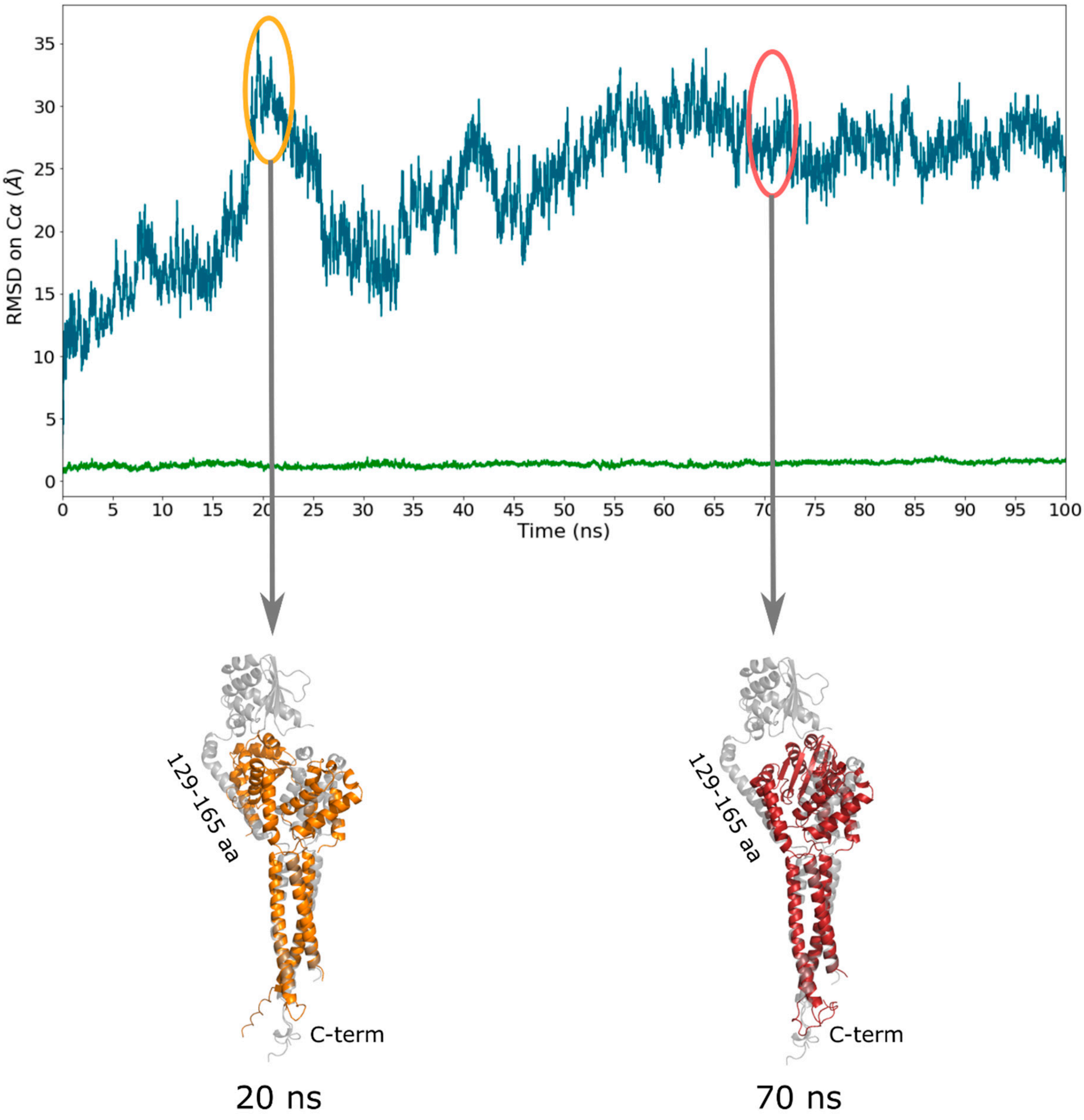

Figure S5. Heterotrimer stability in solution using all-atom MD simulation. On the top, Cα-RMSD is plotted as a function of MD time (ns) for the whole system (blue) and only for the coiled-coil region (green). The RMSD fit was done on Cα atoms of the coiled-coil region, which is defined by residues 171-201 for UbiJ and 49-85 for two chains of UbiK. The first frame of the MD production was used as reference for RMSD calculations. On the bottom, conformational changes in comparison with the reference (grey cartoon) are shown for two MD times, at 20ns (orange cartoon) and at 70ns (red cartoon). Two key flexible regions are labelled such as residues 129-165 for UbiJ and the C-term for the three chains.

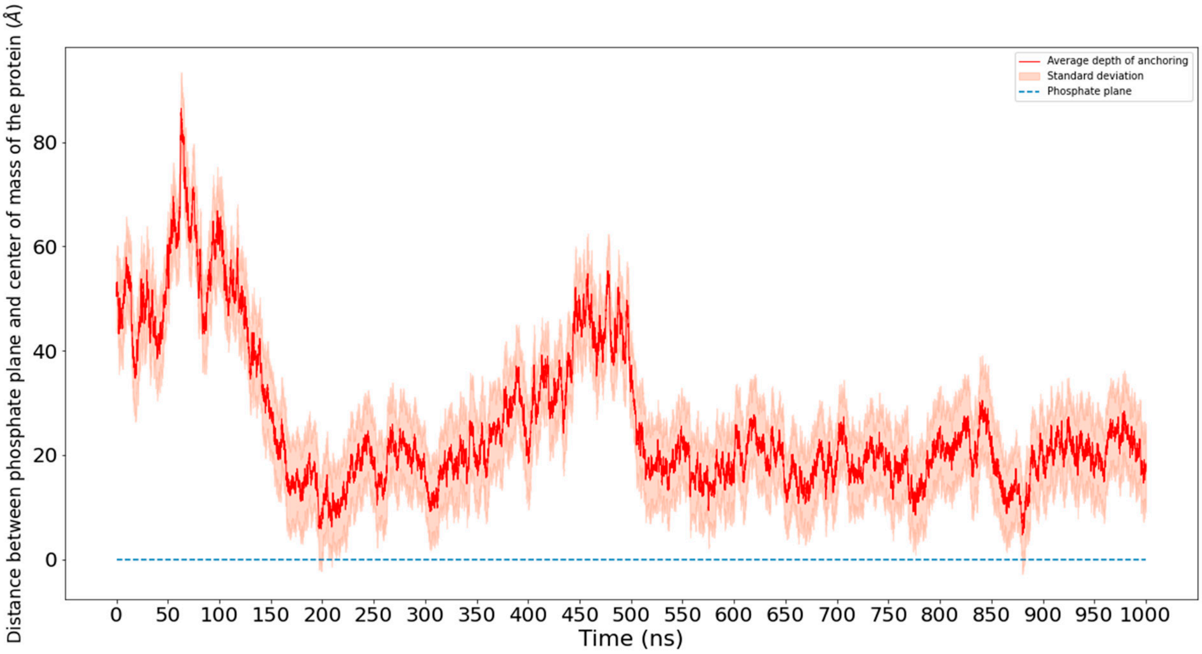

Figure S6. Anchoring of SCP2 into the membrane in CG MD simulation. The plot shows the distance in z-axis between the mass centre of the protein and plane defined by phosphate atoms of the lipids as function of the MD Time. Values close to zero mean that the protein is anchored into the membrane and vice-versa.

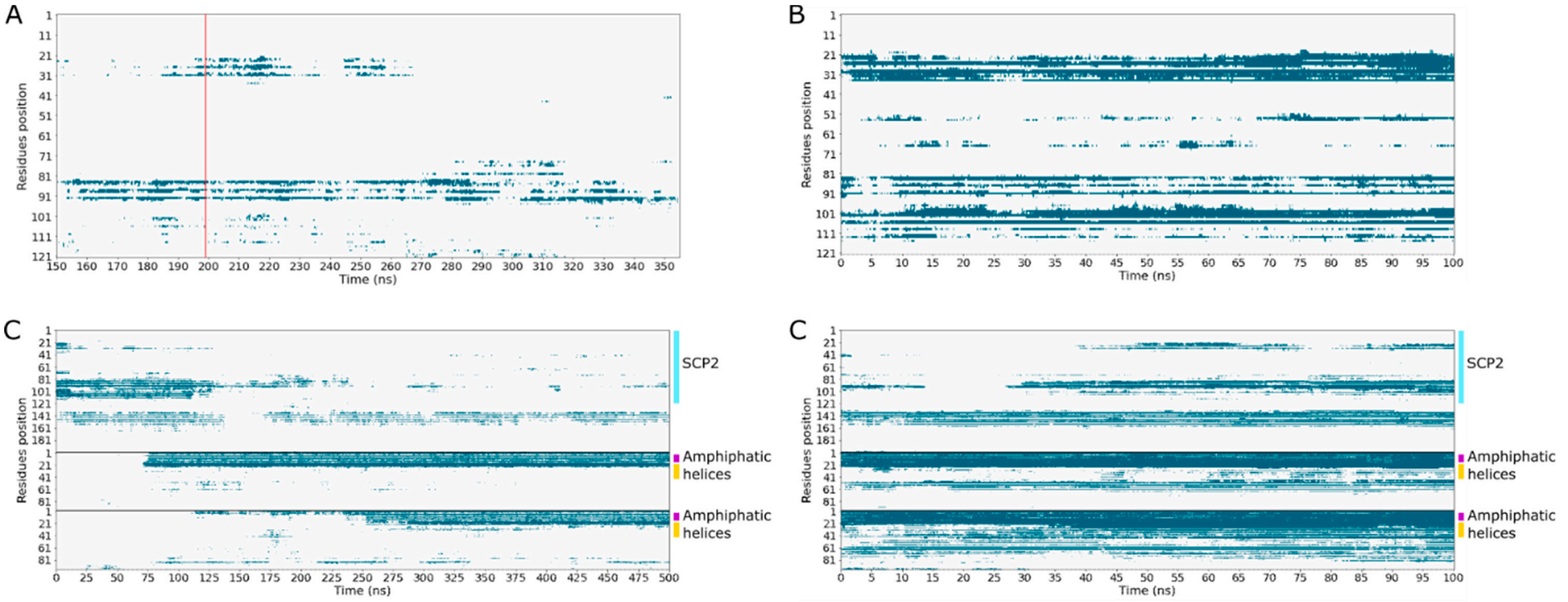

Figure S7. Contact maps from the multiscale MD simulation approach. (A) and (B) correspond to single SCP2 in CG and all-atom respectively, whereas (C) and D) correspond to the heterotrimer in CG and all-atom respectively. For the heterotrimer, key regions such as SCP2 of UbiJ and amphipathic helices in N-ter of UbiK are labelled.

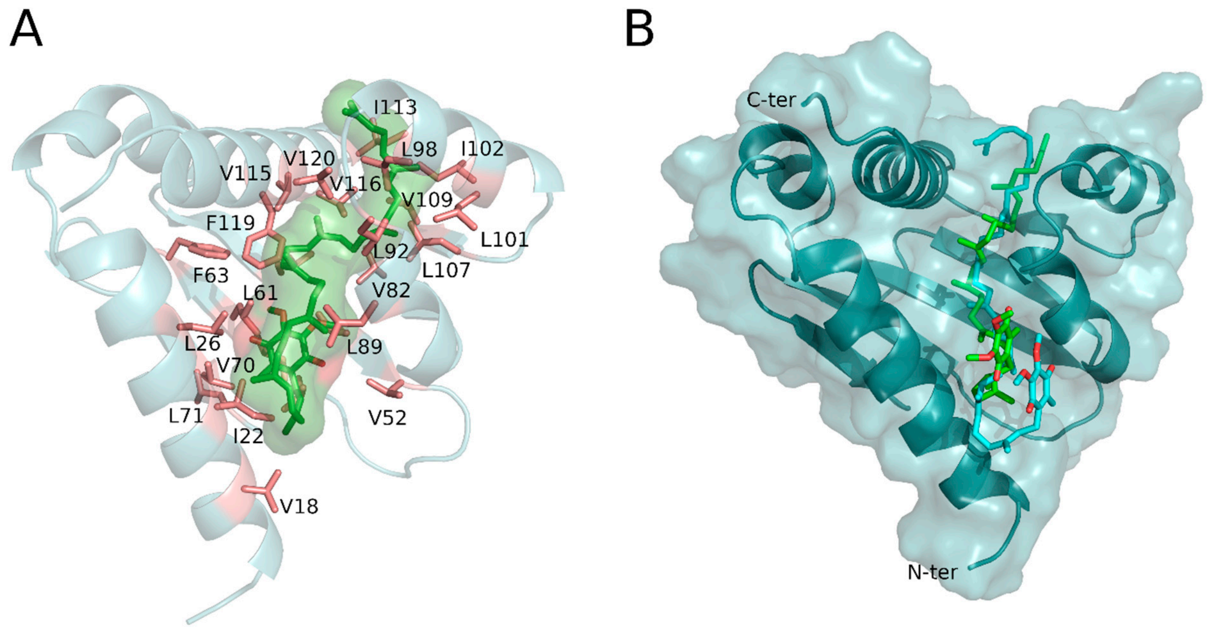

Figure S8. Binding of UQ_8 into SCP2. (A) Initial Manual Docking into SCP2 X-ray structure (PDB code: 6H6O) show in cyan cartoon representation. The hydrophobic residues around 5 Å of UQ_8 are shown in sticks labelled according to the residue name and numbering from PDB. UQ_8 is displayed in green sticks and transparent surface to give an insight of the volume of the groove. (B) UbiJ SCP2 domain is shown in cartoon and transparent surface. UQ_8 from MD simulation and from docking are represented in cyan and green sticks, respectively.

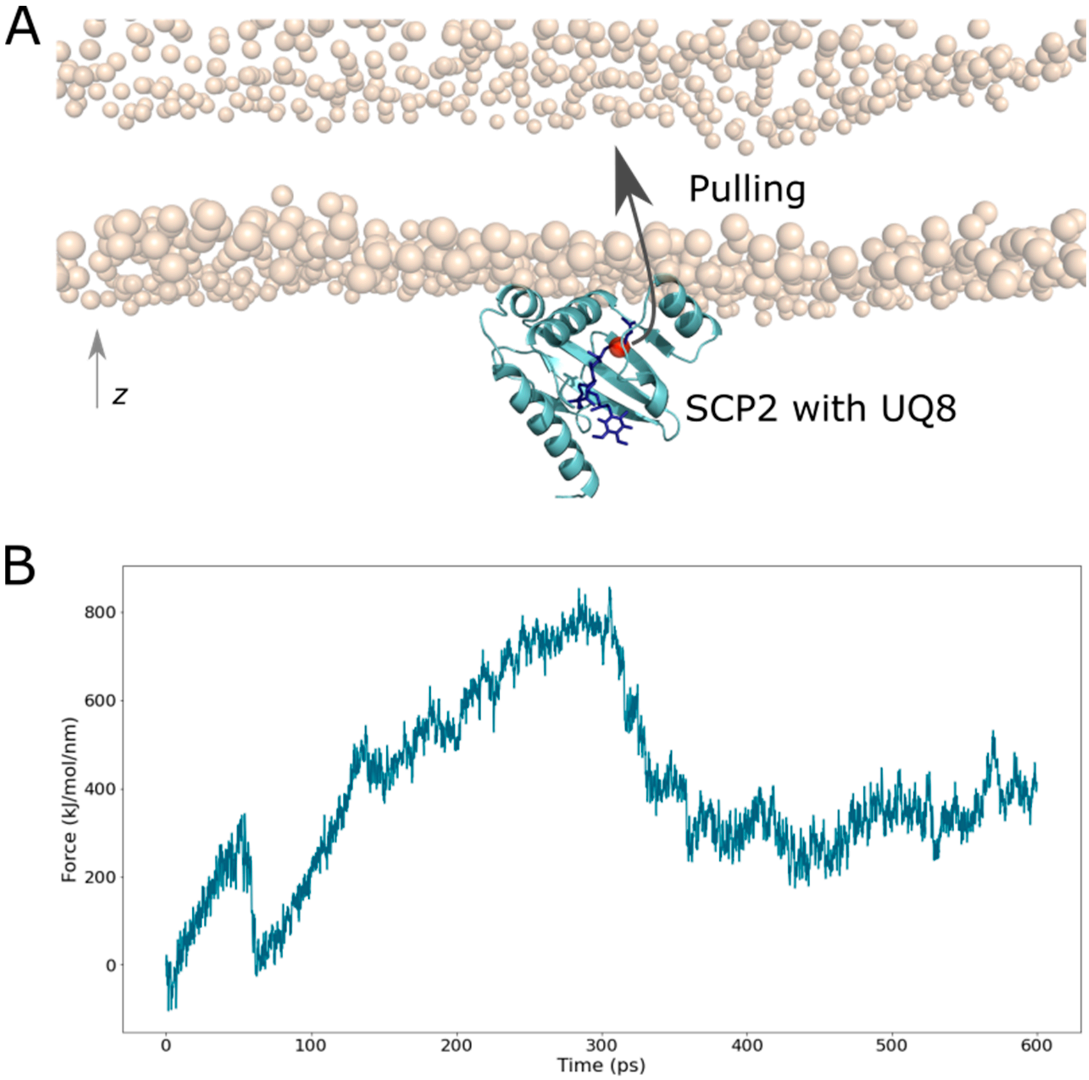

Figure S9. Pulling of UQ_8. (A) Schematic representation of the pulling to release UQ_8 from SCP2 towards the membrane. The carbon (C33) of the UQ_8 hydrophobic tail shown in sphere was used as reference for pulling (B) The force applied for the pulling is plotted as a function of MD time.

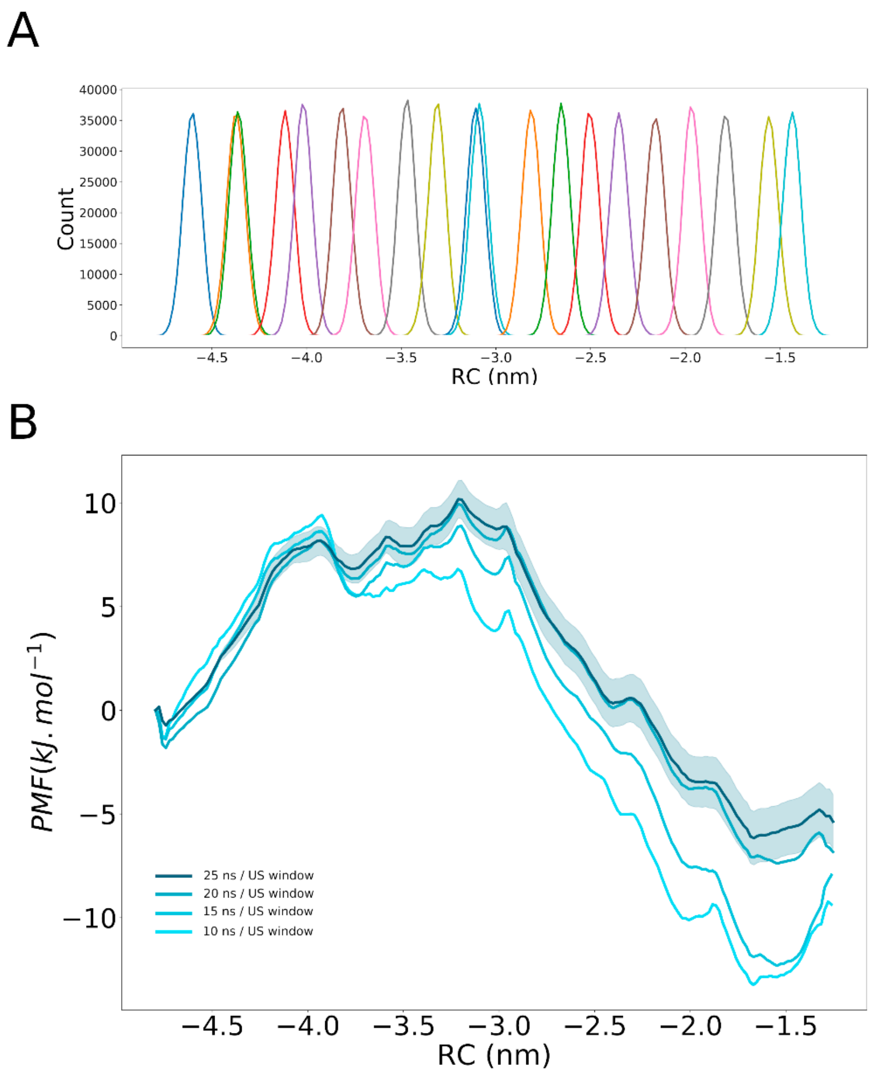

Figure S10. PMF convergence. (A) Umbrella histograms to evaluate the quality of the US sampling. Each gaussian curve corresponds to the sampling of the corresponding US window according to the reaction coordinate (RC) in x-axis. (B) Evolution of the PMF profile as a function of sampling time per US window. The errors on the PMF profile are only plotted for the last sampling (25ns / US window) in transparent. RC is defined in Figure 12 in the Main Text.
